# Successful percutaneous transvenous retrieval of intravascular fractured port catheter: a single center experience

**DOI:** 10.1186/s13019-020-01131-0

**Published:** 2020-05-18

**Authors:** Yahua Li, Jianjian Chen, Zhaonan Li, Huibin Lu, Kewei Ren, Jianzhuang Ren, Xinwei Han

**Affiliations:** 1 Department of Interventional Radiology, The First Affiliated Hospital of Zhengzhou University, Zhengzhou, China; 2 Interventional Treatment and Clinical Research Center of Henan Province, Zhengzhou, China

**Keywords:** Central catheter, Port catheter, Fractured catheter, Interventional radiology, Percutaneous transvenous retrieval

## Abstract

**Background:**

Fractured catheter as a foreign body in situ is a rare complication after port catheter placement. We report a single center’s experience on percutaneous transvenous retrieval of intravascular fractured port catheter and treatment techniques.

**Methods:**

Patients undergoing percutaneous transvenous retrieval of intravascular fractured port catheter from Jan 2010 to Dec 2018 were retrospectively collected. A total of 10 patients (8 females and 2 males) were enrolled in this study. Procedures were performed within 1 day after diagnosis. Two methods of retrieval were considered, direct retrieval by gooseneck snare and guide wire as media to retrieve were used in the procedure.

**Results:**

All the fractured catheters in 10 patients were successfully retrieval by 2 methods, direct retrieval by gooseneck snare(*n* = 6) and guide wire as media of retrieval(*n* = 4). The time interval between port catheter implantation and discovery of catheter fracture was 36.50 ± 42.99(ranged 1 to 146) days. The operation time was 24.10 ± 8.32(ranged 10 to 36) minutes. No immediate procedure related or 1 month follow-up complications occurred in all the 10 patients.

**Conclusion:**

Percutaneous transvenous retrieval of intravascular fractured port catheter is a simple and safe procedure, which maybe recommended as the first choice for patients with fractured port catheter in situ.

## Background

The application of port catheter benefits many patients especially with malignant disease. By administering chemotherapy agents, drugs, blood products and artificial nutrition, this kind of long-term central venous catheter could improve the quality of patient’s life. However, fractured catheter as one of foreign bodies intravascularly is a rare complication after central catheter placement, with an estimate rate of 0–3.1% [1], which is distressing to both patients and interventionalists.

The common approach of port venous catheter implantation is the subclavian vein and internal jugular vein. Fractured catheter would be seen in the subclavian/ internal jugular vein, brachiocephalic vein, superior vena cava, right atrium, right ventricle or pulmonary artery. Although often asymptomatic, potential cardiac perforation, arrhythmias and thromboembolic events are the main reasons for retrieval of this kind of foreign bodies. Management of fractured catheters includes thoracotomy, midsternotomy and endovascular approach. About 94% foreign bodies can be removed by percutaneous transcatheter retrieval technique, a minimally invasive approach [2]. Gooseneck snare, triple loops snare, balloon catheter, basket and forceps were reported to remove fractured catheter successfully [2–9]. Here we share the experience of percutaneous transvenous retrieval of intravascular fractured port catheter and treatment techniques.

## Methods

### Patients

Informed consent was obtained from each patient. Ethics committee approval was obtained for this retrospective study. Patients undergoing percutaneous transvenous retrieval of intravascular fractured port catheter from Jan 2010 to Dec 2018 were retrospectively collected. A total of 10 patients aged from 29 to 84 years were enrolled in this study. All the 10 cases were subcutaneously implanted port catheter via the right internal jugular vein. Fractured catheter was suspected when doing physical examination in 8 patients. One patient presented with pain when receiving liquid transportation and 1 was found during extraction. Chest plain film radiography was taken in order to confirm the diagnosis of catheter fracture and procedures were performed within 1 day after diagnosis.

### Procedure

Patients were placed in supine position. Local anesthesia was delivered with 2% lidocaine, and right femoral or internal jugular vein was chosen to puncture by the Seldinger technique. With the monitoring of ECG and blood pressure, a vascular sheath (8F–14F) was introduced to inferior vena cava or superior vena cava via a guide wire. Angiography was performed by pigtail catheter to show the position of the fractured port catheter. According to the location of the end of a fractured catheter, two methods were used to retrieve them. Since the ends of catheters were free to grasp by gooseneck snare, retrieval of fractured catheters was easily implemented (Method 1). During the procedure, pigtail catheter could use to adjust the position of the end of the catheter when needed. Gooseneck snare was advanced through the free end of a fractured catheter. Then, the segment catheter was encircled with the snare. Finally, gooseneck snare with grasped fractured catheter was pulled out as a unit. While, in other cases where the end of the catheter embedded in vascular tissue or could not grasp after multiple attempts, guide wire would act as media to encompass the body of fractured catheter to retrieve it into vascular sheath (Method 2). During the procedure, a 5F pigtail catheter with guide wire was introduced to the place of fractured catheter. With the assistance of a pigtail catheter, the direction of guide wire tip got changed according to the willing of the operator. Once the guide wire encompassed the middle part of fractured catheter, goose neck snare was utilized to grasp the tip of guide wire. In this situation, the guide wire formed a loop while the fractured catheter was circled in it. The guide wire and gooseneck snare work together as a pulley system to pull out the fractured catheter into the vascular sheath.

## Results

**Fig. 1:**
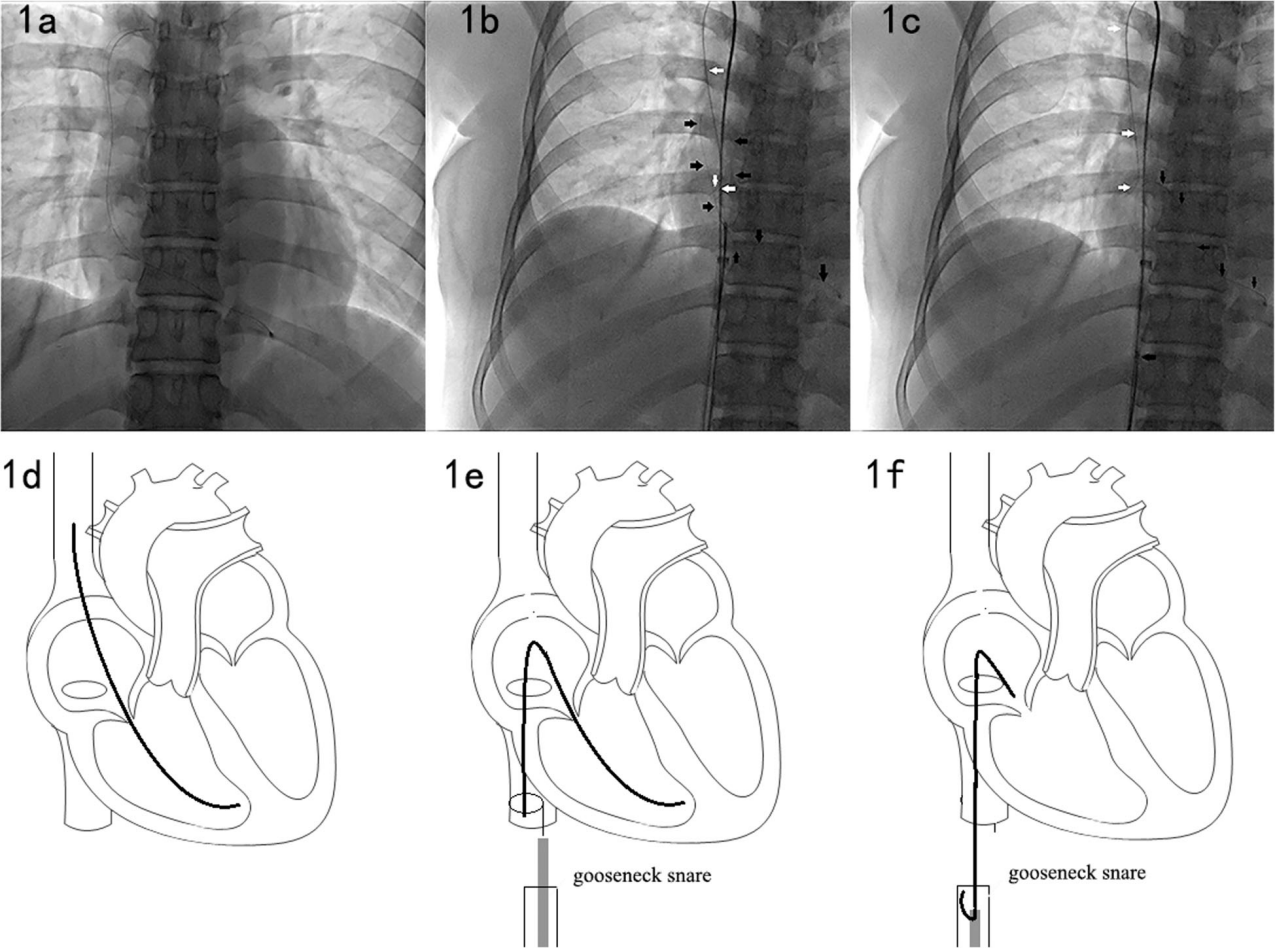
Percutaneous transvenous retrieval of fractured catheter in method 1. **a** Fractured catheter in brachiocephalic vein, superior vena cava, right atrium and right ventricle. **b**, Pigtail catheter and guide wire (white arrow) were used to repositioned the end of fractured catheter (black arrow) to inferior vena cava. Gooseneck snare was used to grasp the end of fractured catheter. **c**, Part of fractured catheter (balck arrow) was retrieval to vascular sheath by gooseneck snare. Pigtail catheter and guide wire still in position. **d**-**e**, The schematic drawings to show the procedure from **a** to **c** in method 1.

**Fig. 2:**
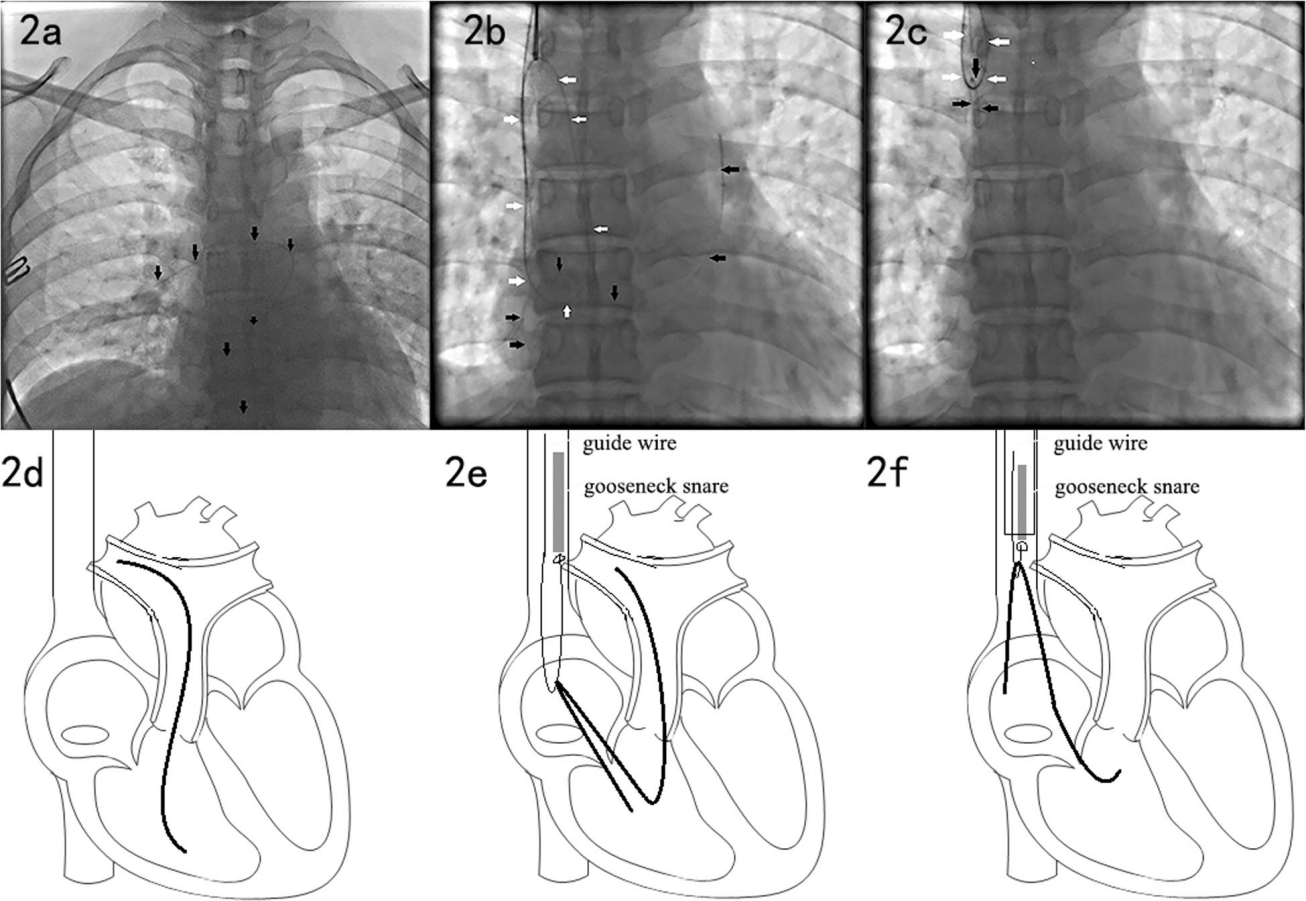
Percutaneous transjugular retrieval of fractured catheter in method 2. **a** Fractured catheter (black arrow) in pulmonary artery and right ventricle. **b** Gooseneck snare was used to help guide wire (white arrow) form a loop where fractured catheter in it. **c** Fractured catheter (black arrow) was successfully retrieval by the guide wire (white arrow) and gooseneck snare. **d**-**e**, The schematic drawings to show the procedure from **a** to **c** in method 2

All the 10 fractured port catheters in 10 patients were successfully removed. In 6 cases with free end of fractured catheter, direct retrieval by gooseneck snare were used. Because it is easy for gooseneck snare to grasp the end of a fractured catheter (Fig. 1). However, the end of fractured catheter in 4 cases were embedded in the vascular tissue and gooseneck snare could not grasp it after several attempts. In this condition, pigtail catheter was used to make the guide wire form a loop to encompass fractured catheter. When the tip of guide wire was grasped by gooseneck snare, the fractured catheter can be locked. By withdrawing guide wire and gooseneck snare together, the fractured catheter was removed (Fig. 2).

All the 10 fractured port catheters were identified in the vein by X-ray plain film radiology. In 4 cases, the port catheter fragment was observed in the right ventricle and pulmonary artery. Three cases with catheter fragment in the right atrium and right ventricle. In 1 case, the fractured catheter exists in brachiocephalic vein, superior vena cava, right atrium and right ventricle. Two cases with fractured catheter in superior vena cava and inferior vena cave, one of them even reached to hepatic vein. The interval between port catheter implantation and discovery of catheter fracture was 36.50 ± 42.99(ranged 1 to 146) days. Procedure was performed within 1 day once the diagnosis was made. All fractured catheters were successfully retrieved by percutaneous transvenous technique, and no fracture happened again. Method 1 was employed in 6 cases and method 2 in 4 cases. The operation time was 24.10 ± 8.32(ranged 10 to 36) minutes. The location of fractured catheter, access vein, procedure time and vascular sheath are shown in Table 1 and Table 2. Neither immediate procedure related nor 1 month follow-up complications occurred in all the 10 patients.

**Table 1:** Clinical characteristics of the patients with fractured catheter

No.	Gender/age	Sign	Interval^a^	Fractured catheter Location	Access vein	Diameter of vascular shealth (F)	Procedure time (min)	Method
1	F/45	no	13	RV-PA	IJV	8	36	2
2	F/40	no	22	RA-RV	RFV	14	28	1
3	F/47	pain	52	RA-RV	RFV	12	29	2
4	F/42	no	45	BV-SVC-RA-RV	RFV	12	20	1
5	F/44	no	19	RA-RV	RFV	12	27	1
6	F/61	no	149	RV-PA	RFV	12	10	1
7	F/29	Pain	35	SVC-IVC-HV	IJV	8	30	2
8	M/47	no	1	RV-PA	RFV	8	16	1
9	M/52	no	28	RV-PA	RFV	12	30	2
10	F/84	no	1	SVC-IVC	RFV	8	15	1

^a^ between implantation and discovery of catheter fracture(days). *BV* brachiocephalic vein, *PA* pulmonary artery, *SVC* superior vena cava, *RA* right atrium, *RV* right ventricle, *HV* hepatic vein, *IJV* internal jungle vein, *RFV* right femoral vein

**Table 2:** Patients’ characteristics

Characteristics	Median or No.
Patients, no.	10
Age, years	49.1 ± 14.7 (29–84)
Pain/no	1/10
Interval^a^	36.5 ± 42.9 (1–149)
Location of catheter	
BV-SVA-RA-RV	1
RA-RV	3
RV-PA	4
SVC-IVC	1
SVC-IVC-HV	1
Access vein	
Internal jugular vein	2
Right femoral vein	8
Procedure time(min)	24.1 ± 8.3 (10–36)
Complications, no	0

^a^ between implantation and discovery of catheter fracture(days)

*BV* brachiocephalic vein, *PA* pulmonary artery, *SVC* superior vena cava, *RA* right atrium, *RV* right ventricle, *HV* hepatic vein, *IJV* internal jungle vein, *RFV* right femoral vein

## Discussion

Port catheter, one of the long-term central venous catheters, plays an important role when treating patients with cancer. Compared with externalized tunneled catheters, this kind of central venous catheter show a lower infection rate and would not affect patients’ daily life [4]. Although most fractured catheter is clinically inconspicuous, severe complications may develop in approximately 71% of the patients with catheter embolism [10].

In a literature review, 94% of endovascular retrieval attempts were successful, with an additional 1.6% retrieved via a combined open/endovascular approach, and only 4% of objects were unretrieved via a minimally invasive approach [2]. Open retrieval is necessary in some cases, mainly about IVC filters, stents, vertebroplasty cement and guide wires. In the early years, Cheng [1] reported 92 cases dislodged central venous catheter retrieved by percutaneous femoral vein technique. The success rate was 97.8% and the complication rate was only 3%. However, with material quality improvement of central venous catheter, the rate of catheter fracture is low. Many documents about fractured catheter are case reports, and case series is rare.

The causes of intravascular foreign bodies were divided into three categories: device defects, inappropriate techniques and patient factors [7]. These three factors are also the reason for fracture of catheter. In our study, the catheter of case 3 was suddenly unavailable during receiving liquid transportation and cannot be felt around subcutaneous port. Obviously, the bad connection between the catheter and port was the main cause of this complication. Inappropriate technique was responsible for case 6 because the catheter was fractured during remove operation. In other 8 patients fractured catheter was found while doing physical examination and they were unaware about the change in port catheter. Device defects are also the main cause in this situation. As for patient factor, compression of the catheter between the clavicle and the first rib (pinch-off syndrome) is the common cause, and usually the catheter placed into the subclavian vein. However, all the catheters in our study were in internal jungle vein, pinch-off syndrome was not reported.

The location of fractured catheter in the cardiovascular system is affected by several factors. The materials and length of catheter, flow pattern of blood and the position of patients when accident happened all contribute to the migration of the catheter. Cheng et al. [1] reported that dislodged catheter mostly in right atrium to inferior vena cava and superior vena cava to right atrium. While, both studies of Bessoud [11] and Peng [6] suggested that pulmonary artery was the most common location of fractured catheter. However, 3 cases in our study were observed in right atrium and right ventricle, 4 cases in right ventricle and pulmonary artery. Which indicates that right ventricle is the common location of fractured catheter. In the study of Cheng [1] and colleagues, the mean port-catheter retention time is 451.6 ± 325.4 days. It is 290 ± 200 days in the study of Bessoud, from central venous device placement to retrieval of a fractured and embolized central venous device catheter. It is only 36.50 ± 42.99 days in our study. It still needs to investigate whether the interval between implantation and discovery of catheter plays a role in the migration of segment catheter after fracture.

The common devices used in the procedure of percutaneous retrieval fractured catheter are vascular sheath, guide wire, pigtail catheter and gooseneck snare. For pigtail catheter or curved catheter could help to reposition the fractured catheter to be more easily snared. Chuang [12] and colleagues reported 23 dislodged port catheter during a 5-year period in their institute, and all the catheters were successfully retrieved with pigtail and snare catheters together. No procedure related complications happened. Wang [13] and colleagues reported percutaneous retrieval of PICC fractures via femoral vein in 6 patients without any related complications encountered. During the procedure, except gooseneck snare and pigtail catheter were used, stone basket catheter still used in one case. In the report of Cheng [1], the devices they used still included flexible triple grasping forceps, floopy guide wire, and multipurpose catheter with self-made loop. However, due to the foreign body in our study are simply the fractured catheter, the use of gooseneck snare and pigtail catheter are enough to retrieve it.

There still exists a voice that leaving the fragments in situ, not all the fractured catheters are indicative for retrieval. Conservative strategy is suitable for moribund patients with difficult–to-extract smaller fragments. The decision of retrieving or not should be made on case-by-case basis. Life expectancy of patients is also need be considered.

The main limitation of our study is the small number of rare cases. Only 10 cases happened in our hospital between Jan 2010 and Dec 2018. Although both retrieval were successful, it also needs further investigation. Another limitation is that we cannot measure the length of the fractured catheter for this retrospectively study. Because the length of the fractured catheter is one of the factors which affect the migration.

## Conclusion

In conclusion, both methods mentioned above are effectively work in procedure. Percutaneous transvenous retrieval of intravascular fractured catheter is simple and safe, which should be recommended as the first choice for patients with fractured catheter intravascularly.

## Data Availability

The datasets used are available from the corresponding author on reasonable request.

## Abbreviations

PICC: Peripherally inserted central catheter
BV: Brachiocephalic vein
PA: Pulmonary artery
SVC: Superior vena cava
RA: Right atrium
RV: Right ventricle
HV: Hepatic vein
IJV: Internal jungle vein
RFV: Right femoral vein
